# Battey’s Operation

**Published:** 1880-11

**Authors:** A. Reeves Jackson


### ARTICLE II.

BATTEY'S OPERATION. Discussion before The Chicago Gynæcological Society, September 17th, 1880. Introduced by A. REEVES JACKSON, M.D. President, DR. BYFORD, in the chair.

In September, 1872, Dr. Robert Battey, of Georgia, published an account of a new surgical operation which he denominated Normal Ovariotomy, and related a case in which he had performed it successfully. The patient was a young lady who was suffering from great impairment of health apparently dependent upon an excessive menstrual molimen, which was not relieved by the usual menstrual flow. All the customary remedies having failed to afford relief, Dr. Battey conceived the idea of artificially and prematurely determining the menopause by removal of the ovaries. This was accordingly done; and the patient recovered with greatly improved health.

This operation was repeated a number of times by its author and others, and the appellation first given to it by the former, being obviously a misnomer, it was re-christened and is now commonly known as "Battey's Operation."

The objects of the procedure are two-fold: (1.) To effect a cure of the various maladies present, by the removal of an ovary viciously or abnormally performing its functions. (2.) By the removal of both ovaries to put an end to ovulation and at once bring about the "change of life."

At the inaugural meeting of the American Gynæcological Society, held in June, 1876, Dr. Battey read a second paper, in which he gave the details of ten cases in which he had performed the operation down to that time; and, as a good deal of misunderstanding was prevalent among the profession as to what cases were proper for it, he stated that it was indicated "in the case of any grave disease which is either dangerous to life or destructive of health and happiness which is incurable by other and less radical means and which we may reasonably expect to remove by the arrest of ovulation, or 'change of life.'" In three of the ten cases reported by Dr. Battey, a single ovary was removed; in the remaining seven both ovaries were removed; the result being eight recoveries and two deaths.

In the following year Dr. Battey presented another paper to the American Gynæcological Society, entitled "Is There a Proper Field for Battey's Operation?" in which he details two more cases of the operation done by him, and specifies the indications for its performance as follows:

1. In those cases of absence of the uterus in which life is endangered or the health destroyed by reason of the deficiency, the removal of the ovaries is at once the hopeful, and the only means of permanent relief.

2. In cases where the uterine cavity or vaginal canal has become obliterated and cannot be restored by surgery, if grave symptoms be present, the removal of the ovaries becomes a last and only resort, and may be hopefully invoked in the case.

3. In cases of insanity, or confirmed epilepsy, dependent upon uterine and ovarian disease, the operation is justifiable as a last resort, and when other means of cure have failed.

4. In cases of long protracted physical and mental suffering, dependent upon monthly nervous and vascular perturbations, which have resisted persistently all other means of cure, the question of a resort to the operation is to be committed to the prudent judgment of the conscientious practitioner in each particular case.

In addition to the foregoing, the operation has been performed for the relief of other conditions; notably, for cases of fibroid tumors of the uterus in which excessive pain and hemorrhage could not be controlled by other means, for the removal of otherwise incurable, prolapsed ovaries, in which grave mental and physical sufferings seemed to depend upon the displacement, as for ovarian dysmenorrhœa.

In the performance of the operation Dr. Battey prefers the vaginal method, while many others, perhaps the majority of operators, prefer the abdominal.

Dr. George J. Englemann, of St. Louis, published a paper in the *American Journal of Obstetrics* for July, 1878, in which he gives the history of three fatal cases of the operation occurring in his own practice, and adds a table showing the result of thirty-six cases, in which the operation had been performed in this country and in Europe. Of this number there were 30.5 per cent. of deaths, and 69.5 per cent. of recoveries. Of those who recovered from the operation there were:

| | |
|---|---|
| Cured | 22.8 per cent. |
| Greatly Improved | 11.4 " |
| Somewhat Improved | 11.4 " |
| Not Improved | 17.2 " |
| Made Worse | 8.6 " |

Now, if we add to the number of those who died, 30.5 per cent. the number of those in whom there was no improvement, 17.2 per cent., and those made worse, 8.6 per cent., we have a total of 56.3 per cent. of failures against 43.7 per cent. of successes; for while in ovariotomy, performed for the removal of cystic or other disease which is destroying the life of the patient, we count it a success when we save that life, a successful case of Battey's operation implies not only the saving of the patient's life, but also the removal or mitigation of her suffering.

Yet, notwithstanding this rather unfavorable showing, I heartily approve of the operation, although I believe it should be confined to a very limited field.

The points of special interest in this subject, and the ones to which I trust it may be the pleasure of the society to limit the conversation, are:

1. The proper indication for the operation.
2. The method of its performance.
3. The results.

DISCUSSION.

DR. NELSON recognized the importance of the operation in certain cases, but the danger to life and the risk of failure should prevent its becoming a frequent resort. It is a matter of importance in operating that the entire ovarian tissue be removed, else the operation might fail in its results.

DR. MILLER asked concerning the indications for the operation: Are we to assume that the operation will cure all diseases which are said to constitute indications for it? For example, in the periodical attacks of epilepsy which some females suffer in ome connection with their menses, are we *to* hope that these attacks will cease after the operation? Is it true that ovarian irritation modifies epilepsy and makes it assume a particular character?

DR. SAWYER inquired as to the effect of this operation upon the sexuality of the subject. Do the feminine characteristics become less prominent in consequence of the contraction?

DR. BARTLETT remarked that the author of the operation had performed it before the Listerian method was in vogue, and now that this method is so well established we may hope for a smaller mortality from the operation. In reply to Dr. Miller, the speaker suggested that epilepsy might recur, after this operation, from habit.

THE PRESIDENT thought that the indications for this operation, which were generally laid down, were largely fanciful. For example, the absence of the uterus, the ovaries being present. He had met with seven such cases, but in no instance was there suffering such as would constitute an indication for this operation. Menstrual disturbances often depend upon the general condition of the patient. Hystero-epilepsy especially was one of these general disturbances. The speaker mentioned the case of a woman who had been under his notice for some years, from whom the ovaries had been removed for the purpose of mitigating the severe hæmorrhages incident to uterine fibroids. The operation had failed utterly, and the fibroids have constantly increased in size. In answer to Dr. Sawyer's question he would say, that in five cases in which the patient was interrogated there was absolutely no diminution in these sexual feelings. Nor was the appearance of the person changed. In this connection he desired to add that in three cases the menstrual flow had never appeared after the operation.

In conclusion, Dr. Jackson said: In deciding upon the proper indications for Battey's operation we should remember that its author has never proposed it as an alternative for any other remedy; nor for any case in which other means have not been persistently and judiciously used and *failed.* He has always held that it should be looked upon as a last resort, more or less hopeful, in cases where there is nothing else to be done,—where the choice is between the operation, and the good it *may* accomplish, on the one hand, and the prolongation of a life of misery and wretchedness, or death, on the other hand. In this respect he has always shown himself exceedingly conservative.

So far as the statistics of the operation show, it has been more successful in relieving the pain and hemorrhages due to the presence of uterine fibrous tumors than in any other class of cases; six of this kind having been reported, with one death.

As regards the mode of performing the operation, I quite agree with the President (Dr. Byford), that the selection of the vaginal or abdominal method should depend upon the circumstances of the case. If the ovaries were low down, or could be easily pressed down so as to be felt through the vaginal vault; and if I could feel tolerably certain that they were not imbedded in inflammatory exudations, or that the neighboring structures had not been the seat of recent inflammation, I should prefer the vaginal route; although ordinarily I should select the abdominal. But, I cannot agree with the opinion expressed by him, that an unsuccessful attempt to operate by the vagina, would seriously complicate the case were we to find ourselves compelled to finish by way of the abdomen. It would not involve much loss of time to enable us to learn whether, from any cause, the operation, per vaginam, were feasible, or otherwise; and we could then proceed to open the abdomen in the usual manner and leave the vaginal opening for subsequent drainage, or close it as we might prefer.

There are two great objections to the vaginal mode. One is the uncertainty as to effecting thorough removal of the ovary, especially when it has become degenerated; and the other is, the very great difficulty in the way of checking hemorrhage in case it should occur. Dr. Battey, who prefers the vaginal operation, and has nearly always chosen it, found it impossible to remove all of the diseased ovarian tissue in four out of twelve cases.

In reply to questions asked, I would say that it is now conceded on all hands that in any case requiring the operation, *both* ovaries should be removed.

The experience of Atlee, Battey, and others, together with that related here to-night by the President, and the facts in a case which occurred to myself, show very clearly, I think, that the womanhood of those who have undergone double ovariotomy is not changed in any respect by reason of the operation.

